# Misconceptions and Barriers to the Use of Hypertonic Saline to Treat Hyponatremic Encephalopathy

**DOI:** 10.3389/fmed.2019.00047

**Published:** 2019-03-15

**Authors:** Juan Carlos Ayus, Michael L. Moritz

**Affiliations:** ^1^Renal Consultants of Houston, Houston, TX, United States; ^2^Division of Nephrology, School of Medicine Irvine, University of California, Irvine, Irvine, CA, United States; ^3^Division of Nephrology, UPMC Children's Hospital of Pittsburgh, University of Pittsburgh School of Medicine, Pittsburgh, PA, United States

**Keywords:** hyponatremia, cerebral edema, hypertonic (3%) saline, encephalopathy, myelinolysis, central pontine, myelinolysis

## Abstract

Hyponatremic encephalopathy is a potentially life-threatening condition with a high associated morbidity and mortality. It can be difficult to diagnose as the presenting symptoms can be non-specific and do not always correlate with the degree of hyponatremia. It can rapidly progress leading to death from transtentorial herniation. Hypertonic saline is the recommended treatment for hyponatremic encephalopathy, whether acute or chronic, yet it is infrequently used. We believe that the main barriers to its use is the perception that hypertonic saline is associated with a significant risk for cerebral demyelination, that it can't be administered through a peripheral IV and that it requires monitoring in the ICU. Two illustrative cases are presented followed by a discussion of how intermittent bolus's of 100−150 ml of 3% NaCl in rapid succession to acutely increase the plasma sodium by 4−6 mEq/L is a safe and effective way to treat hyponatremic encephalopathy, that can be administered through a peripheral IV in a non-ICU setting.

## Key Concepts in the Management of Hyponatremic Encephalopathy

- Hypotonic intravenous fluids should be avoided in hospitalized patients with potential non-osmotic stimuli for vasopressin production in order to prevent hospital-acquired hyponatremia.
- The most consistent clinical features of hyponatremic encephalopathy are headache, nausea and vomiting.
- Neurogenic pulmonary edema can be a presenting manifestation of hyponatremic encephalopathy.
- Patients with acute hyponatremia are not at significant risk for developing cerebral demyelination.
- 3% sodium chloride is the most effective therapy for treating hyponatremic encephalopathy
- Hyponatremic encephalopathy should be treated with repeat 100–150 ml boluses of 3% NaCl in rapid succession with goal of increasing the serum sodium by 5–6 mEq/L in 1–2 h.
- Hypertonic saline can be administered safely through a peripheral vein in a non-ICU setting.

## Introduction

Hyponatremic encephalopathy is a potentially life-threatening condition with a high associated morbidity and mortality. Physicians are most likely to encounter this in post-operative patients (1) or in patients presenting to the emergency department with water intoxication, such as in the setting of exercise associated hyponatremia (2), the recreational drug ecstasy (3) or thiazide diuretic use (4, 5). In the elderly hyponatremic encephalopathy can present with a traumatic hip fracture due to a fall in the home as a consequence a gait abnormality resulting from hyponatremia (6). Hyponatremic encephalopathy can be difficult to diagnose as the presenting symptoms can be non-specific, including headache, nausea, vomiting, lethargy and confusion (7). The symptoms can progress rapidly to coma, seizures, neurogenic pulmonary edema and respiratory arrest, and ultimately lead to death from transtentorial herniation (1). Neurologic symptoms do not always correlate with the degree of hyponatremia, as there are many risk factors for developing hyponatremic encephalopathy including female sex (1), young age (8), hypoxia (9), rapidity of the development of hyponatremia, and underlying CNS disease (10), which can result in hyponatremic encephalopathy in mild to moderate hyponatremia. Physicians must be on the alert for hyponatremic encephalopathy as the symptoms can easily be attributed to another cause, especially when the plasma sodium is > 120 mmol/L.

All experts agree that the definitive treatment for hyponatremic encephalopathy, whether acute or chronic, is hypertonic saline (3% NaCL, sodium 513 mEq/L) (11–13). Consensus guidelines guideline recommend that hypertonic saline be used for the treatment of both acute and chronic symptomatic hyponatremia (12–14). The tonicity of hypertonic saline is sufficiently high that it exceeds the maximum achievable urine tonicity and is capable of increasing the plasma sodium under all circumstances. All studies that have evaluated the use of hypertonic saline have demonstrated its effectiveness in increasing the plasma sodium (4, 15–17). Despite these recommendations and the proven efficacy, hypertonic saline is not frequently used in clinic practice to treat hyponatremia. Well-over 50% of adult patients with severe hyponatremia, plasma sodium < 120 mmol/L, have associated neurologic symptoms (7, 18–20), yet only ~ 2–7% receive treatment with hypertonic saline (17, 19–22), and in many cases it is only administered after other therapies have failed, such as fluid restriction and normal saline. Large studies that have evaluated the management of hyponatremia in hospitalized patients have demonstrated that it is an undertreated condition and that this under-treatment is associated with increased mortality (7–23).

Why isn't hypertonic saline used more often to treat symptomatic hyponatremia when it is an effective and recommended therapy? We believe that there are two primary reasons why physicians are hesitant to use hypertonic saline in the treatment of hyponatremic encephalopathy; (1) they believe that the risks of developing cerebral demyelination associated with using hypertonic saline exceeds the benefits and (2) they believe that hypertonic saline must be administered through a central line in the intensive care unit. We have been consulted on numerous cases of hyponatremic encephalopathy in both children and adults to both assist in the management and to serve as medical experts in patients who have unfortunately died or suffered permanent neurologic injury due to lack of the timely use of hypertonic saline. We repeatedly encounter these two misconceptions as the primary barriers to using hypertonic saline to treat hyponatremic encephalopathy. Below we present two illustrative cases regarding misconceptions related to the use of hypertonic saline to treat hyponatremic encephalopathy followed by a discussion on the appropriate strategies for using hypertonic saline.

## Case #1: Misconceptions Regarding Hypertonic Saline Use and the Development of Cerebral Demyelination

A 28-year-old, healthy female was admitted for acute appendicitis and underwent and appendectomy. Preoperative sodium and renal function was normal. Post-operatively she was administered D5 Ringer's Lactate (Na 130 mEq/L) at 125 ml per hour, with Demerol and Phenergan administered for pain and nausea. Overnight she experienced severe prefrontal headaches with continued nausea and vomiting, which was treated with additional doses of Demerol and Phenergan. The following morning, she was confused and agitated. Her plasma sodium was 123 mEq/L and osmolaity of 255 mOsm/kg with a corresponding urine osmolality of 850 mOsm/kg. A nephrologist was consulted, who felt that her symptoms were consistent with hyponatremia but not symptoms of cerebral edema as she was not actively seizing. It was also felt that hypertonic could result in cerebral demyelination and that using hypertonic saline outweighed the potential benefit. The patient was administered a 1L bolus of 0.9% saline (154 mEq/L) instead to raise the plasma sodium. She then began to seize and had a respiratory arrest. Repeat plasma sodium was 120 mEq/L. She was administered repeated doses of mannitol without neurologic improvement. Hypertonic saline was then administered, and her condition stabilized but she was later declared brain dead and life support was withdrawn.

### Case #1 Discussion

#### Isotonic Fluids Aggravating Hyponatremia in SIAD

**Figure 1:**
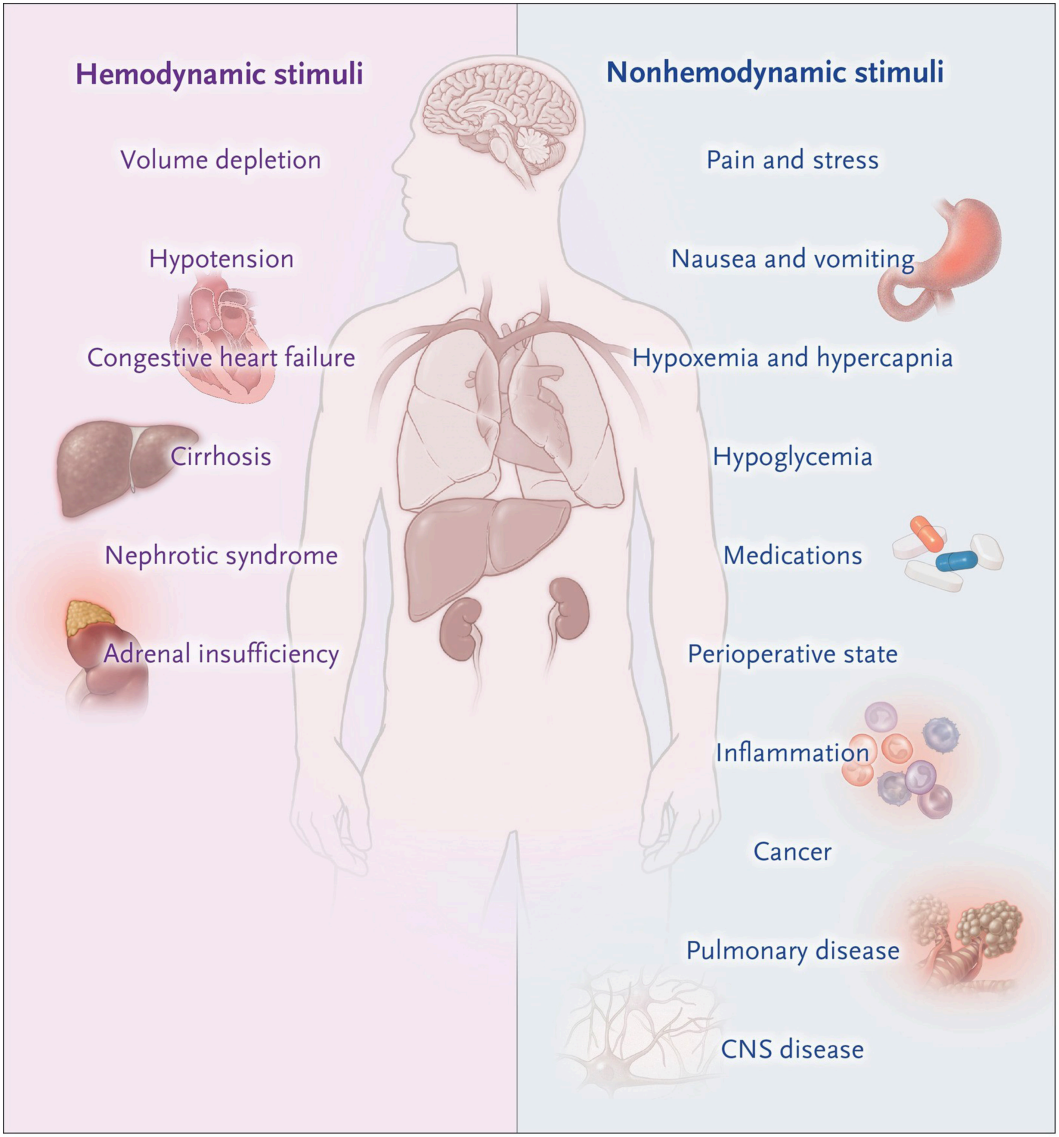
Non-osmotic states of AVP excess. Moritz and Ayus (24).

This patient developed acute post-operative hyponatremia related to a combination of high post-operative AVP levels and the administration of intravenous fluids leading to an SIAD like state with the renal generation of free water due to a physiological natriuresis leading to the excretion of a hypertonic urine (Figure 1) (24, 25). The 1L bolus of 0.9% saline aggravated the hyponatremia due to a powerful natriuresis that ensued (26). Regardless of neurologic symptoms, isotonic fluids should be used with caution in patients with suspected SIAD, as isotonic fluids are known to results in a fall in serum sodium if the urine osmolality is greater than 550 mOsm/kg or the urine tonicity (sodium plus potassium concentration) is great them 154 mEq/L (26). Post-operative nausea and vomiting is a relatively common symptom that does not usually signify hyponatremia. When it occurs in conjunction with severe headaches and is unresponsive to analgesic and antiemetics, hyponatremic encephalopathy should be considered. The physicians recognized that her neurologic symptoms were from hyponatremia, but were under the mistaken belief that headache, nausea, and vomiting were not a sign on cerebral edema and that hypertonic saline was not indicated at this time as there were no active seizures. Headache, nausea and vomiting are the early symptoms of cerebral and a prelude to herniation. Hypertonic saline is indicated at this time to prevent herniation. Radiologic confirmation of cerebral edema is not necessary to initiate therapy in a patient with symptomatic hyponatremia, as it could potentially lead to a life-threatening delay therapy as neurologic symptoms can progress rapidly. If the neurologic symptoms are from hyponatremia, there will be a prompt and marked improvement in symptoms following the partial correction of hyponatremia. They were also under the mistaken believe that this patient was at significant risk for cerebral demyelination if hypertonic saline was used.

#### Cerebral Demyelination and Overcorrection

Cerebral demyelination is a potentially serious complication from the overcorrection of severe and chronic hyponatremia that can lead to permanent neurologic impairment. It is primarily seen in patients with hyponatremia of > 48 h duration and a plasma sodium < 115 mEq/L (12, 27–31). It is infrequently seen in patients with acute hyponatremia or plasma sodium values > 120 mEq/L (12, 31–33). While there are multiple isolated reports of cerebral demyelination occurring in patients with hyponatremia (12), when examined in large series of patients with severe hyponatremia, cerebral demyelination is extremely rare (17, 19, 22). In a report of 1,490 patients with a serum sodium of < 120 mEq/L, the incidence cerebral demyelination was 0.5%, with almost all having multiple other risk factors for developing demyelination, such as alcoholism, hypokalemia, and malnutrition, with the minority receiving 3% NaCl, and most patients making a full neurologic recovery (34). In addition, correction of > 8 mEq/L at 24 h was associated with a lower 30 day mortality then < 8 mEq/L at 24 h (8 vs. 19%). Patients presenting with serum sodium < 115 mEq/L at are particularly high risk for developing overcorrection, independent of what type of therapy is used due to a spontaneous free water diuresis (35). In a series of 11 patients with overcorrection of hyponatremia and median serum sodium of 107 mEq/L, no patients developed cerebral demyelination (35).

This patient was essentially at no risk for developing demyelination as the hyponatremia was acute and the plasma sodium was not very low. Animal studies and clinical observations in humans have demonstrated that cerebral demyelination is a complication from an excessive magnitude of correction of hyponatremia of >25 mEq/L over a 24–48 h period (28, 36, 37). Cerebral demyelination does not appear to be related to an excessive hourly rate of correction as long as the overall magnitude is kept to < 20 mEq/L in the first 48 h of therapy (38–40). The main risk for an overcorrection of hyponatremia is not from the use of hypertonic saline, but rather from renal response to fluid therapy and a spontaneous free water diuresis that occur when the stimulus for AVP release abates. The primary conditions where a brisk free-water diuresis can occur are thiazide-induced hyponatremia, water intoxication, volume depletion, adrenal insufficiency following replacement therapy and dDAVP-induced hyponatremia following dDAVP withdrawal (41). In patients with severe and chronic hyponatremia, dDAVP has been used successfully to curtail a free water diuresis and prevent overcorrection of hyponatremia, as well as to therapeutically re-lower the serum sodium following an inadvertent overcorrection of hyponatremia (42, 43). Cerebral demyelination can occur with any therapy used to correct severe and chronic hyponatremia, including normal saline and V2 antagonists, if there is an excessive correction of hyponatremia (22–44).

There are risk factors other than the correction of hyponatremia that can place a patient at risk for developing cerebral demyelination such as severe liver disease (28), hypokalemia (29, 31), thiazide diuretic use, alcoholism, malnutrition (45), hypophosphatemia (46), and hypoxia (9). Most reported patients with cerebral demyelination have had one or more of these risk factors. Patients with these risk factors have been reported to have cerebral demyelination even in the absence of hyponatremia (47–49). This patient had none of these risk factors for developing demyelination.

## Case #2: Misconceptions Regarding The Route and Setting Necessary to Administer Hypertonic Saline

A 21-year-old female presents to the emergency department via ambulance unresponsive and in respiratory distress. She is frothing at the mouth and has diffuse crackles on auscultation with a room air saturation of 80%. Her pupils are unequal and minimally responsive. She is not seizing. Her plasma sodium is 119 mEq/L. The prior evening, she was at a night club dancing and had taken multiple tablets of the recreational drug ecstasy (3,4-methylenedioxymethamphetamine [MDMA]) and was drinking profusely. Her boyfriend found her unresponsive in the morning and called 911. The Emergency room physician recognized this as ecstasy-associated hyponatremia presenting with neurogenic pulmonary edema as a manifestation of hyponatremic encephalopathy. Two hundred Milliliters of hypertonic saline was ordered to be administered emergently. The pharmacy would not dispense the hypertonic saline as it was hospital policy that hypertonic saline could only be administered in the intensive care unit through a central line and could not be administered via a peripheral IV. Plans were made to transfer the patient to the intensive care unit. They were informed that there would be a delay due a high census in the ICU. She was then intubated and transferred to the ICU 4 h later. During transport she develops a tachyarrhythmia followed by a cardiac arrest that failed attempts at resuscitation.

### Case #2 Discussion

#### Peripheral Administration of 3% Sodium Chloride

**Figure 2:**
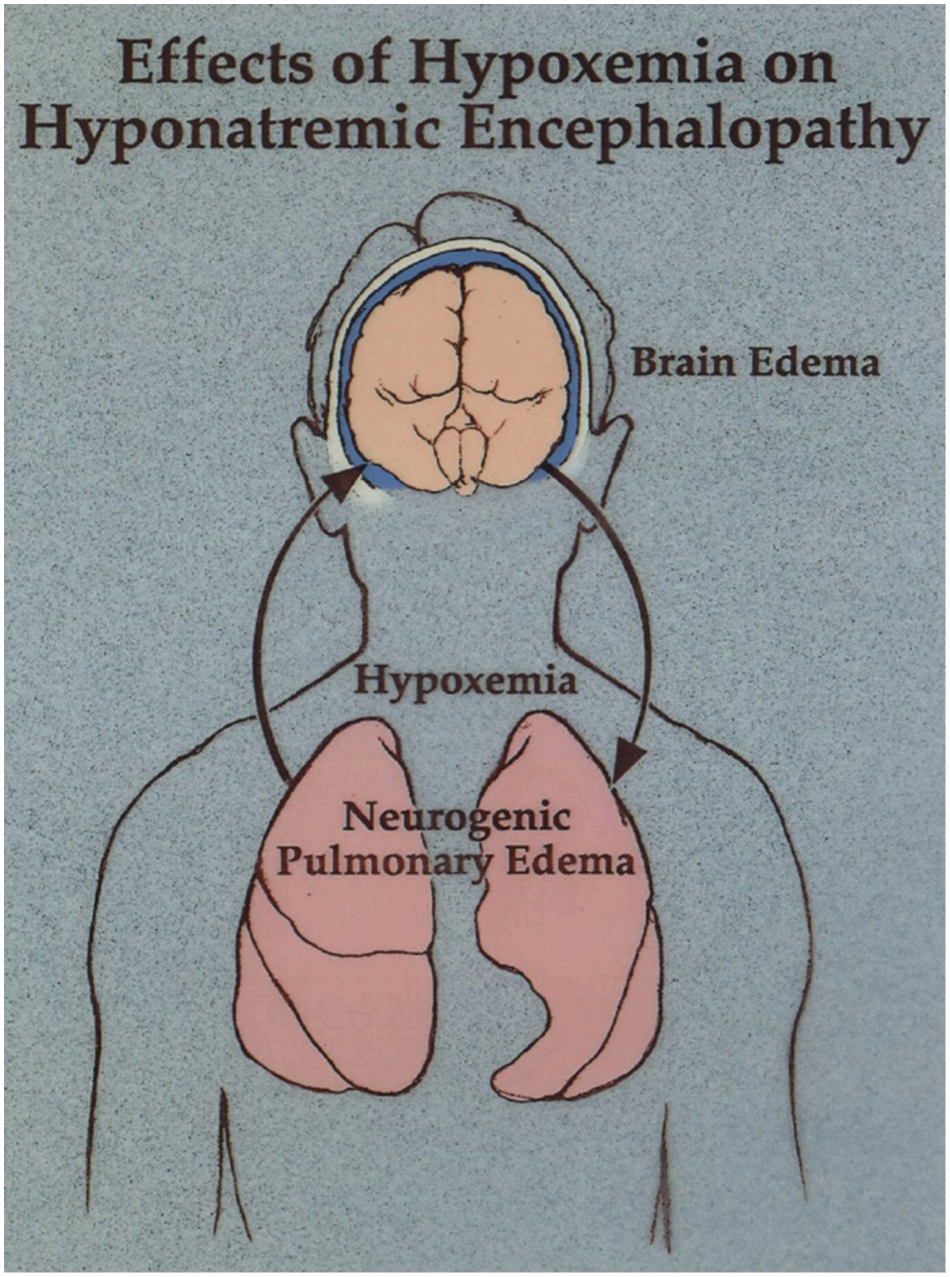
Neurogenic pulmonary edema as a manifestation of hyponatremic encephalopathy. Moritz and Ayus (11).

In this case the physicians recognized that patient had neurogenic pulmonary edema as a manifestation of hyponatremic encephalopathy (2, 50). Hyponatremia produces cytotoxic cerebral edema, which in turn leads to a neurogenic pulmonary edema. Pulmonary edema leads to hypoxia, which impairs brain cell volume regulation resulting in a vicious cycle of worsening cerebral edema and pulmonary edema (Figure 2). They realized that hypertonic saline was necessary to reverse this condition, but they were prohibited from treating her in a timely fashion because of a hospital policy which restricted the administration of hypertonic saline through a peripheral IV and unfortunately lead to her untimely death. There is a widespread misconception that 3% sodium chloride can cause phlebitis and regional necrosis when administered through a peripheral vein. This is based on literature demonstrating a relationship between phlebitis and prolonged infusions of hypertonic total parenteral nutrition over days to weeks (51). This does not apply to short term infusions of hypertonic saline to acutely raise the plasma sodium to treat hyponatremic encephalopathy. We treated 71 consecutive episodes of hyponatremic encephalopathy presenting to the emergency department with a unified protocol of 500 ml of 3% sodium chloride administered over 6 h through a peripheral vein and there were no complications related to the infusion (4). Studies in both children and adults have demonstrated the safety of 3% sodium chloride administered through a peripheral vein with a < 10% incidence of minor infusion infiltrations usually associated with prolonged infusions, without any serious complications (52–55).

The primary reason why a patient with hypertonic saline should be transferred to the intensive care unit is not because of the monitoring required to administer hypertonic saline, but rather to monitor the patient's neurologic symptoms closely as patients with hyponatremic encephalopathy can have a respiratory arrest and require intubation and mechanical ventilation. There is a report of 35-year-old adult with acute hospital-aggravated hyponatremia where the physicians recommended treating with intravenous 3% NaCl, yet they had to resort to using hourly oral sodium chloride tablets as their hospital prohibited the use of 3% NaCl in the non-ICU setting and there was a city-wide public health emergency preventing transfer to the ICU (56). These pharmacy restrictions on 3% NaCl are not consistent with the lack of restrictions on hypertonic 20% mannitol (1,100 mOsm/L) and 8.4% sodium bicarbonate (2,000 mOsm/L) which both have an osmolality greater the than 3% NaCl (1,027 mOsm/L).

#### The Hypertonic Saline Bolus: A Safe and Effective Approach to Prevent Overcorrection of Hyponatremic Encephalopathy

Our group has reported on over 30 years of experience studying the use of hypertonic saline to treat hyponatremic encephalopathy. We have used this in both children and adults, in patients with acute and chronic hyponatremia, in patients presenting at a medical tent or emergency department and in patients with hospital-acquired hyponatremia (1, 2, 4, 6, 8, 10, 11, 15, 28, 50, 57–61). We have not encountered cerebral demyelination as a complication of hypertonic saline in any cases that we have personally treated. We do recognize that the indiscriminate use of hypertonic saline and prolonged infusions of hypertonic saline without appropriate monitoring can produce neurologic injury from an excessive correction of hyponatremia. Also, patients with severe hyponatremia, < 120 mEq/L, are at risk for overcorrection of hyponatremia regardless of what therapy is used if the stimulus for vasopressin production abates and a free water diuresis. There are also varying opinions regarding safe limits to correcting hyponatremia (12, 62). In 2005, we introduced a simple approach of using small repeated intermittent boluses of hypertonic saline in order to achieve a rapid and controlled increase in serum sodium to treat hyponatremic encephalopathy, that conformed to the various opinions on the safe limits of correcting hyponatremia and minimizes the risk of inadvertent overcorrection (57).

**Figure 3:**
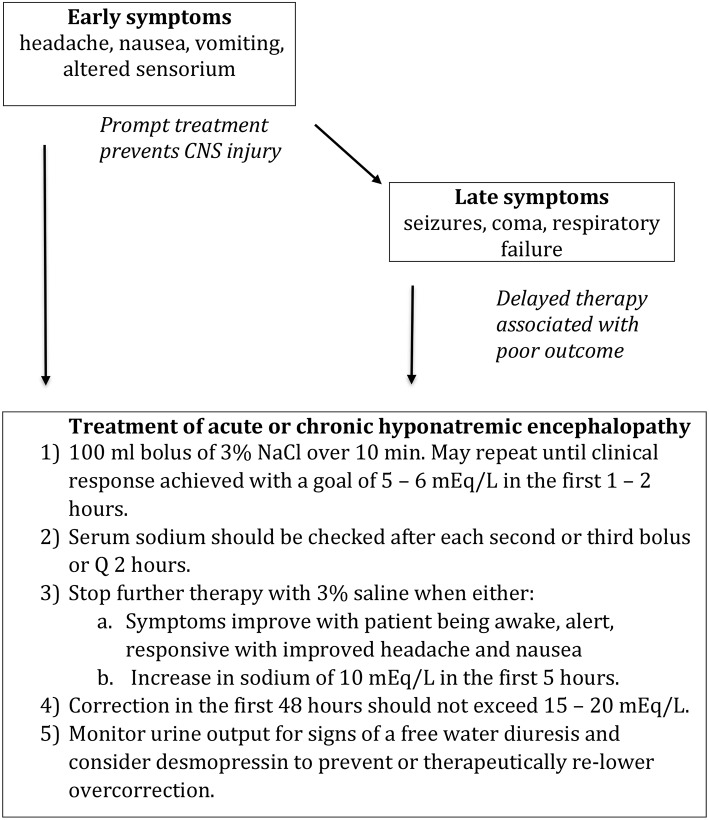
Treatment of hyponatremic encephalopathy. Modified from Achinger and Ayus (63).

Our approach is to treat any patient with suspected hyponatremic encephalopathy, with either moderate or advanced symptoms, child or adult, with a 2 ml/kg bolus of 3% NaCl. The bolus could be repeated 1–2 times in sequential fashion if symptoms persisted (Figure 3) (60, 63). A single bolus would result in, at most, a 2 mEq/L acute rise in serum sodium, which could quickly reduce brain edema. In most cases, a 4–6 mEq/L acute rise in plasm sodium will begin to reverse the neurologic symptoms, and failure to show some clinical improvement following an acute elevation in serum sodium would suggest that the patient is not suffering from hyponatremic encephalopathy. The bolus approach can be given safely through a peripheral IV and can be used in a non-ICU setting. It would be effective regardless of the etiology of hyponatremia, serving as a volume expander in hypovolemic hyponatremia and is sufficiently hypertonic to increase the serum sodium in euvolemic hyponatremia from SIADH. It also does not require the use of complicated formulas, which have been demonstrated to be inaccurate as they assume a closed system and do not account for the renal response to therapy (64–66). Other therapies such as 0.9% NaCl, 1.8% NaCl, mannitol, urea, oral sodium, and vaptans, cannot be recommended as first-line therapies to treat hyponatremic encephalopathy as they do not reliably increase the serum sodium or reverse the neurologic symptoms.

Our approach has now been adopted for the treatment of hyponatremic encephalopathy by expert panels including the International Exercise-Associated Hyponatremia Consensus Development Congress (14) and the European Clinical Practice Guidelines (12).

According to the recent European Clinical Practice Guidelines, hypertonic saline solution is recommended for the treatment of hyponatremic encephalopathy regardless of whether it is acute or chronic, whether the symptoms are moderate or severe, or if the degree of hyponatremia is moderate (125–129 mmol/l) or profound (< 125 mmol/l). They assessed the risk of brain edema to outweigh the risk of cerebral demyelination. Their recommendations call for symptomatic patients to have the plasma sodium raised by a target of 5 mmol/l within 1 h with hypertonic saline, while limiting the increase in serum sodium concentration by 10 mmol/l during the first 24 h and an additional 8 mmol/l during every 24 h thereafter until the serum sodium concentration reaches 130 mmol/l. (12). The guidelines' recommendation for hypertonic saline solution were based on 9 case series that varied widely in regard to the setting, symptoms, severity, duration, and therapy used to treat hyponatremic encephalopathy. Their recommendations for limits on serum sodium were based on 54 cases of cerebral demyelination published since 1997. They found that the 87% of cases had the plasma sodium concentration increased ≥ 12 mmol/l during the first 24 h and ≥ 20 mmol/l during the first 48 h. According to the guidelines, most case reports used a total of 500 ml of 3% sodium chloride solution.

Our initial recommendations limited the bolus to a maximum volume of 100 ml. The recent European Clinical Practice Guidelines recommend a 150 ml bolus for adult, which we have no objection to (12). The ideal bolus volume and number of boluses required has not been determined in clinical trials, and it may prove that an even larger bolus volume could be safe and effective.

Our group conducted a prospective observational study comparing IV sodium chloride compared to fluid restriction for the treatment of chronic hyponatremic encephalopathy (mean sodium 111 mmol/l) in 53 patients (6). All patients treated with IV sodium chloride prior to a respiratory arrest had neurologic recovery without any apparent evidence of demyelination despite an increase in plasma sodium of 22 mmol/l in the first 48 h, whereas all patients treated with fluid restriction, primarily due to the concern of developing demyelination, died or developed permanent neurologic injury (6). We subsequently conducted a prospective trial treating 71 episodes of hyponatremic encephalopathy (mean sodium 114 mmol/l) presenting to the emergency department with a uniform protocol of 500 ml of 3% sodium chloride over 6 h (4). Most of these cases were presumably due to chronic hyponatremia and ninety-seven percent had a reversal of neurologic symptoms without developing clinical evidence of cerebral demyelination on long term follow (4). This data is highly suggestive that hypertonic saline is safe and effective for the treatment of both acute and chronic hyponatremic encephalopathy. It needs to be emphasized though, that the risks of cerebral demyelination are very real in the high-risk patient with known risk factors such as severe and chronic hyponatremia, Na < 115 mEq/L, with alcoholism, liver disease, hypokalemia or malnutrition. The lack of cerebral demyelination in our case series despite serum sodium correction > 20 mEq/L if 48 h does not me that we recommend a sodium correction of that degree. Controlling the absolute magnitude of hyponatremia can be difficult though and in the vast majority of cases as slight overcorrection of guideline recommendation does not result in demyelination.

## Summary

Hypertonic saline is an underused therapy to treat hyponatremic encephalopathy due to the misconceptions that it can produce cerebral demyelination, that it can't be administered through a peripheral IV and that it requires monitoring in the ICU. Using repeated intermittent bolus's of 100–150 ml of 3% NaCl in rapid succession to acutely increase the plasma sodium by 4–6 mEq/L is a safe and effective way to treat hyponatremic encephalopathy. It can be administered through a peripheral IV and in a non-ICU setting. This approach limits the possibility of an inadvertent overcorrection of hyponatremia from excessive administration of hypertonic saline. If a spontaneous free water diuresis occurs in a patient with severe and chronic hyponatremia, dDAVP can be administered to curtail the water diuresis and prevent and inadvertent overcorrection of hyponatremia.

## Author Contributions

JA conceived of the manuscript. MM drafted the manuscript. MM and JA edited the manuscript.

### Conflict of Interest Statement

The authors declare that the research was conducted in the absence of any commercial or financial relationships that could be construed as a potential conflict of interest.
